# Motor and Gaze Behaviors of Youth Basketball Players Taking Contested and Uncontested Jump Shots

**DOI:** 10.3389/fpsyg.2018.00706

**Published:** 2018-05-14

**Authors:** Mariëtte J. J. van Maarseveen, Raôul R. D. Oudejans

**Affiliations:** ^1^Faculty of Sports and Nutrition, Amsterdam University of Applied Sciences, Amsterdam, Netherlands; ^2^Department of Human Movement Sciences, Amsterdam Movement Science, Vrije Universiteit Amsterdam, Amsterdam, Netherlands; ^3^Institute of Brain and Behavior Amsterdam, Amsterdam, Netherlands

**Keywords:** visual search strategy, representative design, perception, motor behavior

## Abstract

In this study, we examined the effects of a defender contesting jump shots on performance and gaze behaviors of basketball players taking jump shots. Thirteen skilled youth basketball players performed 48 shots from about 5 m from the basket; 24 uncontested and 24 contested. The participants wore mobile eye tracking glasses to measure their gaze behavior. As expected, an approaching defender trying to contest the shot led to significant changes in movement execution and gaze behavior including shorter shot execution time, longer jump time, longer ball flight time, later final fixation onset, and longer fixation on the defender. Overall, no effects were found for shooting accuracy. However, the effects on shot accuracy were not similar for all participants: six participants showed worse performance and six participants showed better performance in the contested compared to the uncontested condition. These changes in performance were accompanied by differences in gaze behavior. The participants with worse performance showed shorter absolute and relative final fixation duration and a tendency for an earlier final fixation offset in the contested condition compared to the uncontested condition, whereas gaze behavior of the participants with better performance for contested shots was relatively unaffected. The results confirm that a defender contesting the shot is a relevant constraint for basketball shooting suggesting that representative training designs should also include contested shots, and more generally other constraints that are representative of the actual performance setting such as time or mental pressure.

## Introduction

In sports, the ability of performers to use information from the environment to select and execute an appropriate action is essential to high-level performance (Williams and Ericsson, 2005; Williams et al., 2011). This ability is based on an accurate and efficient relationship between perceptual and motor processes, termed the “perception–action coupling” (Gibson, 1979; Michaels and Beek, 1995). Due to the dynamic and fast-paced nature of sport settings, opportunities for action emerge and disappear as individuals interact with their environment. Performers need to learn to continuously adapt their behavior to the changing task constraints, and consequently the design of appropriate task constraints is a major issue in research and learning perceptual-motor skills.

Representative design is a concept initially proposed by Brunswik (1956) and states that in research tasks should be created in such a way that the task constraints represent the behavioral setting to which the results are intended to be generalized (Dicks et al., 2009; Pinder et al., 2011). Recent studies show significant changes in movement and gaze behavior under different experimental task constraints accompanied by varying degrees of perception–action coupling. Findings of meta-analyses of perceptual-cognitive skill in sports have shown that expertise effects are more apparent under *in situ* task constraints than in less representative conditions (Mann et al., 2007; Travassos et al., 2013). For example, Dicks et al. (2010) showed that soccer goalkeepers made more penalty saves and fixated earlier on the ball and for longer periods of time in an *in situ* condition where actual interception was required compared to responding to a video simulation involving limited movement. Such findings have major implications for the creation of experimental and learning designs in sports (Pinder et al., 2011; Travassos et al., 2012).

Even when using natural sports performance settings, ensuring that the task constraints are representative is not easy since small changes in task constraints can lead to significant changes in performance outcomes and movement responses (Hristovski et al., 2006; Pinder et al., 2011; Travassos et al., 2012). In invasion sports, immediate opponents offer relevant constraints on action possibilities. A defender (almost) by definition has considerable perturbing effects upon the actions of an attacker. Therefore, in research and training, tasks requiring the performer to execute a skill against an opponent may provide a more representative design of the actual performance setting (Brunswik, 1956; Pinder et al., 2011; Gorman and Maloney, 2016). However, there is only a limited number of studies comparing contested and uncontested conditions (for examples, see Rojas et al., 2000; Hughes et al., 2010; Rivilla-Garcia et al., 2011; Orth et al., 2014; Gorman and Maloney, 2016; Klostermann et al., 2017). These studies generally reveal that players change their movement behavior when facing a defender in various sports (e.g., Rojas et al., 2000; Hughes et al., 2010; Rivilla-Garcia et al., 2011).

The influence of a defender on motor performance of basketball shots has been demonstrated by the findings from empirical research (Rojas et al., 2000; Gorman and Maloney, 2016). For example, Rojas et al. (2000) found that when professional basketball players perform a jump shot against a defender trying to contest the shot, the speed, release height, and release angle of the ball were increased. These are all likely adaptations to reduce the chance of the opponent blocking the ball. Similarly, Gorman and Maloney (2016) found that a defender led to faster shot executions, longer jump times, and longer ball flight times. Furthermore, these changes in motor execution were accompanied by a decrease in shooting accuracy of over 20%. However, the shooting accuracy was based on just six trials in each of five different shot types, meaning that hitting one shot more or less resulted in a change in shooting accuracy of 16.7%. Nonetheless, even at the elite level of the NBA, the proximity of a defender influences shooting accuracy. When NBA players have a wide open shot (i.e., the defender is more than 6 ft away), the average shooting accuracy of three-point shots is 38.1%; for open shots (4–6 ft), this is 35.4%; for tight shots (2–4 ft) 31.2%, and for very tight shots (0–2 ft), this is 26.4% (NBA Advanced Stats, 2016–2017 data^1^).

One possible cause for the reduced shooting accuracy against an opponent may be the visual control of the basketball shot. Visual control of basketball shooting has been examined in the static task of free throw shooting and in more dynamic tasks like taking jump shots. Vickers (1996) examined the gaze behavior of basketball players during static free throws, and found that experts’ duration of the final fixation before the initiation of the movement was significantly longer than for lesser skilled performers. This phenomenon called quiet eye is defined as “the final fixation or tracking gaze that is located on a specific location or object in the task space within 3° of visual angle (or less) for a minimum of 100 ms. The onset of the quiet eye occurs prior to a critical final movement in the task and the offset occurs when the gaze deviates off the object or location by more than 3° of visual angle for a minimum of 100 ms, therefore the quiet eye can carry through and beyond the final movement of the task” (Vickers, 2016, p. 1–2). The quiet eye period reflects the time needed to set the parameters of the movement to be executed (preprogramming; Vickers, 1996; Williams et al., 2002), and suggests an open-loop process for controlling the shooting movements (Ripoll et al., 1986; Vickers, 1996).

However, a number of studies by Oudejans et al. (2002, 2005; de Oliveira et al., 2006, 2007, 2008) challenged this finding and found evidence for online visual control of the basketball shot. Using the dynamic task of basketball jump shooting, Oudejans et al. (2002) found that shooting with late vision (i.e., vision occluded until the last ± 350 ms before ball release) was as good as shooting with full vision, while early vision (i.e., vision occluded during the last ± 350 ms) resulted in a decrease in performance. These results imply that the final shooting movements were controlled by continuous pick-up and use of visual information until ball release, and shows that the last ± 350 ms before ball release are necessary and sufficient for accurate shooting. This was confirmed in the study by de Oliveira et al. (2008) who examined the final fixation on the rim in basketball jump shooting. They used a slightly different definition of the final fixation on the rim than that of quiet eye as (i) the onset of the final fixation on the rim does not have to be prior to initiation of the final shooting movement (e.g., the extension of the shooting arm in basketball shooting), as long as it is prior to ball release, and (ii) the offset is never later than ball release because after ball release the shooter cannot control the ball anymore, implying that vision after ball release is useless for movement control of that shot. The gaze results corroborate the view that basketball shooting is largely controlled online by vision, that is, visual information is picked up and used during movement execution.

To date, the influence of a defender on the visual control of basketball shots has only been examined by Klostermann et al. (2017). They compared quiet-eye behavior of intermediately skilled and highly skilled basketball players in contested vs. uncontested game situations, and found that the absolute quiet eye duration did not significantly differ between contested and uncontested shots. Still, a longer relative quiet eye duration was found for the contested compared to the uncontested shots. However, as relative quiet eye duration is defined as absolute quiet eye duration divided by the total movement time, this merely reflected a change in total movement time from 1178 ms in the uncontested condition to 519 ms in the contested condition rather than a change in absolute quiet eye duration. Furthermore, in the “uncontested game situation,” shots were taken from one position after making a dribble, while in the contested game situation, jump shots could be made after a dribble or pass in three vs. three small sided game situations (Klostermann et al., 2017, p. 3). Actions preceding the jump shot (pass or dribble) may influence the shooting accuracy (Oudejans et al., 2012a). In addition, data collection lasted until participants reached six hits and six misses leading to a wide range of number of shot attempts varying from 12 to 56, and a differential basis for calculating shooting accuracy. Finally, the method of analysis of gaze behavior was unclear. The duration of phases and the starting moments of a phase were used interchangeably. Also, the onset and offset of quiet eye were calculated as relative values in relation to the beginning of the final extension of the shooting arm. This is practically less interesting than the timing of the final fixation in relation to the moment of ball release, as that is the moment at which control over the ball ends (cf. Rojas et al., 2000; Oudejans et al., 2002; Gorman and Maloney, 2016).

The purpose of the present study was to examine the influence of a defender contesting the shot on (motor) performance and gaze behavior of talented youth players taking basketball jump shots. The accuracy of the shots were recorded as well as several measures of movement and gaze behavior, including shot execution time, jump time, ball flight time (similar to the study of Gorman and Maloney, 2016), and the duration and timing of the final fixation on the rim prior to ball release. It was hypothesized that an approaching defender would decrease the shooting accuracy and would cause changes in movement variables that are required to prevent the shot from being blocked by the defender (Rojas et al., 2000; Gorman and Maloney, 2016). In line with earlier studies, we expected faster shots, higher jumps, and longer ball flights in the contested compared to uncontested shots. As for gaze behavior we expected shorter relative, but more importantly also absolute final fixations on the rim indicative of hampered visual control of the shot.

## Materials and Methods

### Participants

A total of 13 talented female basketball players participated in this study [a number comparable to similar studies on basketball shooting, Gorman and Maloney, 2016 (*n* = 12), Klostermann et al., 2017 (*n* = 15 and 8), Oudejans et al., 2002 (*n* = 10), and Rojas et al., 2000 (*n* = 10)]. The average age of the participants was 16.8 years (*SD* = 1.8 years). The participants were all enrolled in the national basketball talent program and national youth team, and had an average of 8.0 years (*SD* = 1.8 years) of playing experience. Their average seasonal statistics were 44.5% for field goals, 18.5% for three-point shots, and 59.3% for free throws. The experiment was approved by the scientific and ethical review committee of the Faculty of Behavioral and Movement Sciences of the Vrije Universiteit in Amsterdam and all participants gave their written informed consent prior to the experiment; parental consent was provided for participants younger than 16 years.

### Equipment

**FIGURE 1:**
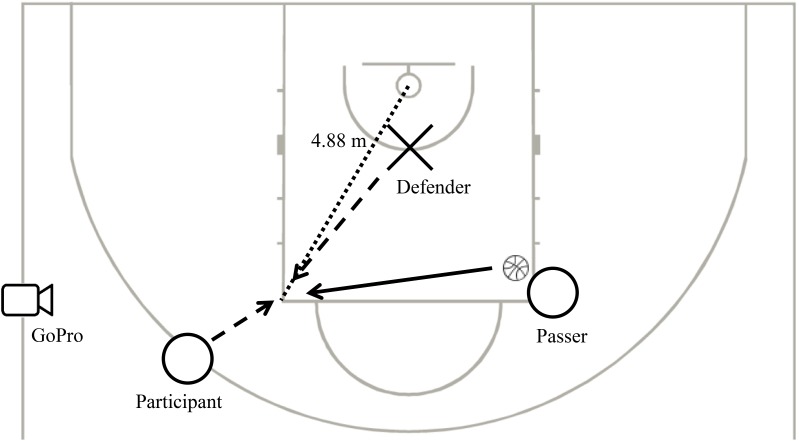
Schematic top view of the experimental setup. Shooting from the left side is depicted. Participant (the shooter) steps in and receives the ball from the passer and takes a shot. In the contested condition, the defender (cross) steps in toward the participant to contest the shot. In the uncontested condition the defender stays in her original location.

All trials were recorded with a GoPro camera (Hero 3, black edition, GoPro, Inc., United States) that was positioned on the side line of the court and in line with the free throw line (**Figure 1**). The SensoMotoric Instruments eye tracking glasses (SMI; Teltow, Germany; binocular, 30 Hz) were used to record the gaze behavior of the participants. The glasses were either connected to a mobile recording unit which was carried in a waist bag (with data storage on a hard disk in the recording unit, and a wireless live view on a laptop) or via a 5-m-long usb-cable to a laptop. In both cases, the participants were able to move freely. Prior to testing, the eye tracking glasses were calibrated using a three-point calibration and the calibration was checked and adjusted if needed prior to each series of 12 shots. The test took place at the regular training facilities of the national basketball talent program. Official FIBA regulation court, basket, and women’s basketball (size 6) were used.

### Procedure and Design

Participants were assigned to matching pairs by the head coach based on playing position, height, and skill level. The participants performed a brief warm-up including some shooting drills prior to testing. The test consisted of a total of 24 shots in both the contested and uncontested condition, and these comprised 12 shots from the left and 12 shots from the right side of the court. The test conditions and playing side of the court were counterbalanced across participants. The test took approximately 20 min to complete per pair of participants.

Every trial started with a signal from one of the experimenters. The participant moved toward the elbow (i.e., corner of the free throw line) to receive the pass from the experimenter who was positioned on the other elbow (see **Figure 1**). The participant was instructed to shoot immediately after receiving the pass. In the contested condition, the defender ran out to defend the shot, but only after the starting signal given by the experimenter. This allowed sufficient time for the defender to reach the participant and contest the shot. Defenders were instructed to contest the shot without making actual contact with the participant. They made a so-called close-out with one arm and hand up in the air. In the uncontested condition, the defender remained standing on the restricted arc. The participants and defenders were instructed to perform the test in a game-like manner with the same speed and intensity as they would normally show. If the pass to the participant was reckoned to have considerable disadvantages for the participant or the defender made contact with the participant, the trial was repeated.

### Data Analyses

Shooting accuracy was determined for both the contested and uncontested condition by summing the number of successful shot attempts and dividing it by the number of test trials. The recordings of the SMI eye tracking glasses were synchronized with the video footage of the GoPro camera using Adobe Premiere Pro. These synchronized video files were analyzed frame by frame for the duration of each trial using Dartfish. Identical to Gorman and Maloney (2016), three movement variables were extracted from the video recordings: shot execution time, jump time, and ball flight time. Shot execution time was measured from the moment when the ball first touched either of the shooter’s hands, to the moment when the ball first lost contact with the shooter’s shooting hand during the execution of the shot (i.e., moment of ball release). Jump time was measured from the moment when both of the shooter’s feet first left the floor to go up for the jump shot, to the moment when either of the shooter’s feet first resumed contact with the floor after the ball was shot. Ball flight time was measured from the moment when the ball left the shooter’s hand (i.e., moment of ball release) to the moment when the ball first touched (or would have touched) either the rim or backboard.

The synchronized video footage was also used to analyze gaze behavior of the participants. A fixation was defined as gaze maintained on any location for a period equal to or in excess of 100 ms or three sequential frames (cf. Williams and Davids, 1998; Savelsbergh et al., 2002; Vaeyens et al., 2007a,b). We determined the fixations on the locations rim and defender, and were especially interested in the *final* fixation on the rim before ball release [following the same definition as de Oliveira et al. (2008), which deviates in some regards from the definition of quiet eye, see section “Introduction”]. Relative final fixation duration and relative occlusion duration were calculated by dividing the absolute values by the shot execution time. The onset and offset of the final fixation on the rim were calculated in relation to ball release (e.g., 100 ms means 100 ms before ball release). As visual control ends at the moment of ball release, the offset could not occur after ball release. In case gaze was still fixated at the rim at the moment of ball release, the offset was coded as 0 ms.

The video footage was randomly assigned to two experimenters who coded the movement variables and gaze behavior. A total of 48 randomly selected trials were coded by both experimenters to assess inter-observer reliability, and it was found that on average the ICC = 0.98, *p* < 0.001 for the movement variables and κ = 0.91, *p* < 0.001 for gaze behavior, indicating excellent agreements (Hallgren, 2012).

#### Statistical Analyses

Shooting accuracy in the contested and uncontested condition was analyzed using a paired samples *t*-test. The three movement phases, and the seven variables of gaze behavior were analyzed using separate repeated measures ANOVAs with the factors condition (contested vs. uncontested) and outcome (hits vs. misses). For the factor side of the field (left vs. right), analyses revealed no significant effects. Therefore, this factor was excluded from the analyses reported in this paper, also because this factor was not of principal interest. For all ANOVAs, significant main and interaction effects were followed up by Bonferroni corrected pairwise comparisons. Effect sizes were reported as partial eta squared ($\eta_{p}^{2}$), and the significance level was set at 0.05.

As the results revealed that there were large individual differences in response to the approaching defender, we were interested to examine this further. We therefore, *a posteriori*, created two sub-groups based on the shooting accuracy because six participants showed lower shooting accuracy in the contested condition than in the uncontested condition, and six participants showed higher shooting accuracy in the contested than in the uncontested condition. We therefore classified them as the worse and better group, respectively. One player showed identical shooting accuracy in the contested and uncontested conditions. Therefore, she could not be classified as worse or better and was excluded from the *a posteriori* group analyses. We realize that this procedure of creating groups is neither common nor desirable. However, we believe that the averaging out that occurred conceals relevant findings. In the end, the final test in this study is not about differences in shooting accuracy between these groups but the accompanying differences in gaze behavior. We will first present the results for the group as a whole after which we will also present the analyses with the *a posteriori* created groups.

## Results

### Shooting Accuracy

The mean shooting accuracy of the participants was 52.2% (*SD* = 8.1%) in the uncontested condition and 51.3% (*SD* = 15.0%) in the contested condition, *t*(12) = 0.199, *p* = 0.85, *r* = 0.06, ns, giving the impression that an approaching defender did not affect shooting accuracy.

### Movement Phases

The movement phases are displayed in **Table 1**. The analysis of shot execution time showed a significant main effect of condition, *F*(1,12) = 39.87, *p* < 0.001, $\eta_{p}^{2}$ = 0.77, revealing that contested shots were performed significantly faster than uncontested shots. The main effect for outcome and the condition x outcome interaction effect were not significant, both *F*s < 0.24, *p*s > 0.63. For jump time, also a significant effect for condition was found, *F*(1,12) = 32.00, *p* < 0.001, $\eta_{p}^{2}$ = 0.73, indicating that the jump time of contested shots was longer than for uncontested shots. The main effect for outcome and the condition x outcome interaction effect were not significant, *F*(1,12) = 4.05, *p* = 0.07, $\eta_{p}^{2}$ = 0.25, and *F*(1,12) = 0.04, *p* = 0.84, $\eta_{p}^{2}$ = 0.00, respectively. The ball flight time also differed as a function of condition, *F*(1,12) = 9.76, *p* < 0.05, $\eta_{p}^{2}$ = 0.45, with a significant longer ball flight time for contested shots than uncontested shots. There was also a significant effect of outcome, *F*(1,12) = 6.80, *p* < 0.05, $\eta_{p}^{2}$ = 0.36, indicating that the ball flight time of hits was shorter than of misses. The condition x outcome interaction effect was not significant, *F*(1,12) = 0.16, *p* = 0.70, $\eta_{p}^{2}$ = 0.01.

**Table 1:** Mean (*SD*) duration of the movement phases and gaze behavior variables for hits and misses in the uncontested and contested conditions. For definitions of the phases and durations, we refer to the text (see section “Data Analyses”).

	Uncontested			Contested		
	Hits	Misses	Average	Hits	Misses	Average
**Movement phases**					
Shot execution time (ms)	898 (107)	894 (96)	896 (100)	819 (93)	816 (72)	817 (82)
Jump time (ms)	250 (63)	247 (61)	249 (61)	278 (53)	276 (54)	277 (52)
Ball flight time (ms)	990 (55)	998 (57)	994 (55)	1014 (60)	1027 (69)	1021 (64)
**Gaze behavior variables**					
Final fixation duration (ms)	433 (246)	453 (202)	443 (221)	369 (181)	360 (206)	364 (191)
Rel. final fixation duration (%)	48 (26)	51 (22)	49 (24)	45 (22)	44 (26)	45 (23)
Onset final fixation duration (ms)^†^	555 (209)	565 (170)	560 (187)	503 (165)	469 (202)	486 (182)
Offset final fixation duration (ms)^†^	122 (140)	111 (131)	116 (133)	133 (120)	109 (113)	121 (115)
Occlusion (ms)	127 (86)	119 (89)	123 (86)	117 (70)	114 (73)	116 (70)
Rel. occlusion (%)	14 (10)	13 (10)	14 (10)	15 (9)	14 (9)	14 (8)
‘Gaze on defender (ms)	0 (0)	8 (28)	4 (20)	61 (70)	49 (84)	55 (76)

*^†^Calculated relative to ball release with positive numbers indicating occurrence prior to ball release.*

### Gaze Behavior

Gaze behavior of the participants is displayed in **Table 1**. The ANOVA for final fixation duration revealed a significant main effect for condition, *F*(1,12) = 14.554, *p* < 0.05, $\eta_{p}^{2}$ = 0.559; the final fixation was shorter in the contested condition than in the uncontested condition. The main effect for outcome and the condition x outcome interaction effect were not significant, both *F*s < 0.78, *p*s > 0.39.

For the relative final fixation duration, a significant main effect for condition was found, *F*(1,12) = 7.00, *p* < 0.05, $\eta_{p}^{2}$ = 0.37. Again a shorter relative final fixation duration was found in the contested condition than in the uncontested condition. The main effect for outcome and the condition x outcome interaction effect were not significant, both *F*s < 0.80, *p*s > 0.39.

For final fixation onset, a significant main effect for condition was found, *F*(1,12) = 14.78, *p* < 0.05, $\eta_{p}^{2}$ = 0.55; the final fixation onset was later in the contested condition than in the uncontested condition. The main effect for outcome and the condition x outcome interaction effect were not significant, *F*s < 1.01, *p*s > 0.33. For final fixation offset, no significant main nor interaction effects were found, *F*s < 2.99, *p*s > 0.11.

The analyses of the occlusion duration and the relative occlusion duration did not reveal significant effects, all *F*s < 2.13, *p*s > 0.17.

For the fixation duration on the defender, a significant main effect for condition was found, *F*(1,12) = 8.87, *p* < 0.05, $\eta_{p}^{2}$ = 0.42, with participants fixating longer on the defender in the contested condition than in the uncontested condition. The main effect for outcome and the condition x outcome interaction were not significant, *F*s < 0.46, *p*s > 0.51.

### *A Posteriori* Analyses

As mentioned, to further accommodate the individual differences in performance response to the approaching defender, we *a posteriori* created the two sub-groups, the worse and better groups. Their mean (and *SD*) shooting accuracy, movement phases, and gaze behavior variables for the uncontested and contested conditions are displayed in **Table 2**. We first checked the creation of the subgroups using an ANOVA on the shooting accuracy. A significant condition x group interaction effect was found, *F*(1,10) = 21.11, *p* < 0.001, $\eta_{p}^{2}$ = 0.68. In line with how the groups were created, the participants with worse performance showed lower shooting accuracy in the contested condition than in the uncontested condition, *p* < 0.05, whereas the participants with better performance showed higher shooting accuracy in the contested compared to the uncontested condition, *p* < 0.05. The shooting accuracy of the worse and better groups was not significantly different in the uncontested condition, *p* = 0.12, but it was in the contested condition, *p* < 0.05. The main effect for condition, *F*(1,10) = 0.11, *p* = 0.75, as well as the main effect for group, *F*(1,10) = 1.86, *p* = 0.20, were not significant.

**Table 2:** Mean (*SD*) shooting accuracy, duration of movement phases, and gaze behavior variables for the participants with worse and better performance (in the contested compared to the uncontested condition), in the uncontested and contested conditions. For definitions of the phases and durations, we refer to the text (see section “Data Analyses”).

Group	Worse (*n* = 6)		Better (*n* = 6)	
Condition	Uncontested	Contested	Uncontested	Contested
Shot accuracy (%)	56.3 (9.8)	41.0 (13.8)	48.6 (5.0)	61.8 (9.3)
**Movement phases**			
Shot execution time (ms)	896 (92)	823 (67)	890 (125)	812 (109)
Jump time (ms)	261 (68)	287 (54)	239 (67)	270 (61)
Ball flight time (ms)	985 (55)	1017 (69)	999 (64)	1015 (69)
**Gaze behavior variables**			
Final fixation duration (ms)	440 (265)	332 (205)	385 (137)	357 (169)
Rel. final fixation duration (%)	48.3 (27.7)	40.1 (24.5)	44.1 (17.8)	44.2 (20.6)
Onset final fixation duration (ms)^†^	501 (233)	429 (189)	574 (115)	533 (159)
Offset final fixation duration (ms)^†^	61 (64)	96 (84)	188 (164)	175 (138)
Occlusion (ms)	113 (107)	117 (93)	147 (67)	126 (55)
Rel. occlusion (%)	12.7 (11.3)	14.3 (10.9)	17.0 (8.2)	15.7 (7.0)
‘Gaze on defender (ms)	0 (0)	131 (80)	0 (0)	67 (76)

*^†^Calculated relative to ball release with positive numbers indicating occurrence prior to ball release.*

For shot execution time, jump time, and ball flight time, the main and interaction effects involving group were not significant, all *F*s < 0.64, *p*s > 0.44. Thus, all participants (worse and better) showed shorter shot execution time, longer jump time, and longer ball flight time for contested than uncontested shots (see original analyses).

The ANOVA for final fixation duration revealed a significant main effect for condition, *F*(1,10) = 14.34, *p* < 0.05, $\eta_{p}^{2}$ = 0.59; the final fixation was shorter in the contested condition (*M* = 345 ms, *SD* = 180 ms) than in the uncontested condition (*M* = 412 ms, *SD* = 203 ms). The condition x group interaction effect was marginally significant, *F*(1,10) = 4.87, *p* = 0.052, $\eta_{p}^{2}$ = 0.33. A shorter final fixation duration was found in the contested condition than in the uncontested condition for the participants with worse performance, *p* < 0.05, but not for the participants with better performance, *p* = 0.29. The main effect for group was not significant, *F*(1,10) = 0.02, *p* = 0.90.

For the relative final fixation duration, a significant main effect for condition was found, *F*(1,10) = 7.39, *p* < 0.05, $\eta_{p}^{2}$ = 0.43, as well as a significant condition x group interaction, *F*(1,10) = 8.08, *p* < 0.05, $\eta_{p}^{2}$ = 0.45. A shorter relative final fixation duration was found in the contested condition than in the uncontested condition for the participants with worse performance, *p* < 0.05, but not for the participants with better performance, *p* = 0.93. The main effect for group was not significant, *F*(1,10) = 0.00, *p* = 0.99.

For final fixation onset, a significant main effect for condition was found, *F*(1,10) = 9.18, *p* < 0.05, $\eta_{p}^{2}$ = 0.48. The final fixation onset was later in the contested condition (*M* = 481 ms, *SD* = 175 ms) than in the uncontested condition (*M* = 538 ms, *SD* = 179 ms). The main effect for group and the condition x group interaction effect were not significant, *F*s < 0.75, *p*s > 0.41. For final fixation offset, no significant main effect for condition nor group was found, *F*s < 2.32, *p*s > 0.16, whereas the condition x group interaction was marginally significant, *F*(1,10) = 3.68, *p* = 0.08, $\eta_{p}^{2}$ = 0.27. By tendency, only the participants with worse performance showed earlier final fixation offset in the contested condition compared to the uncontested condition, *p* = 0.08 (*p* = 0.48 for the participants with better performance).

The analyses of the occlusion duration and the relative occlusion duration did not reveal significant effects, all *F*s < 3.66, *p*s > 0.09.

For the fixation duration on the defender, a significant main effect for condition was found, *F*(1,10) = 19.25, *p* < 0.05, $\eta_{p}^{2}$ = 0.66, with participants, as mentioned, fixating longer on the defender in the contested condition than in the uncontested condition. The main effect for group and the condition x group interaction were not significant, *F*s < 2.05, *p*s > 0.18.

## Discussion

The purpose of this study was to examine the influence of an approaching defender on (motor) performance and gaze behavior of talented youth players taking basketball jump shots. Thirteen skilled youth basketball players performed shots from elbow distance under both contested and uncontested conditions, while concurrently wearing mobile eye tracking glasses to measure their gaze behavior. As expected, shot execution was faster, jump time was longer, and ball flight time was longer in the contested compared to the uncontested condition. These changes in movement execution were similar to the findings of Gorman and Maloney (2016) and seem to reflect the participants’ attempts to adapt their movement to the approaching defender (Rojas et al., 2000; Gorman and Maloney, 2016; Klostermann et al., 2017). In practical terms, players shot faster, jumped higher, and propelled the ball with a higher arc toward the basket. These are all likely adaptations to reduce the chance of the opponent blocking the ball (Rojas et al., 2000). This confirms that the direct proximity of a defender influences motor behavior, and that this is an important consideration when designing representative shot trainings or study designs (see also Davids et al., 2008; Renshaw et al., 2010; Pinder et al., 2015).

However, in contrast to earlier findings (Gorman and Maloney, 2016; Klostermann et al., 2017), the behavioral changes in our study were not accompanied by an overall decline in shooting accuracy. Instead, it appeared that different participants were differentially affected by the presence of a defender, with six participants showing lower and six participants showing higher shooting accuracy in the contested compared to the uncontested condition. This suggests that not all players were successful in adapting their shot to the presence of the defender. The performance of some participants was actually hindered under influence of a defender, while other participants were able to successfully adapt to the varying task constraints and even managed to perform better. It is possible that shooting against a defender resulted in distraction from the main shooting task in some of the players. This is supported by the findings on gaze behavior as the *a posteriori* analyses revealed that the overall effects that were found for gaze behavior (i.e., shorter final fixations in the defended condition and earlier offset) were in fact only present for the participants who shot worse with a defender. This suggests that these participants missed out on the relevant visual information to control their shot, and this could explain the decrease in their performance when facing a direct defender.

In contrast, the participants with better performance did not show differences in gaze behavior between the contested and uncontested conditions. The duration of their final fixation on the rim was not affected and apparently remained sufficiently long. In addition, the timing of this final fixation did not change. Looking for an explanation for why they managed to actually improve their performance we can only speculate, for instance, that the defender led to better concentration and focus on the task. Alternatively, perhaps the defender provided an additional informational frame of reference (in the periphery) providing a better basis to perceive the distance to the rim and control the shot movements accordingly (Greenwood et al., 2016). This would fit the findings of Greenwood et al. (2016) who found that the umpire in cricket may provide a vertical reference point for the bowlers to regulate their run-ups. Future research is needed to determine whether the defender might provide such an informational constraint in basketball shooting. Overall, the results of the current study confirm the importance of the duration and timing of the final fixation for accuracy in far-aiming tasks like the basketball jump shot (Oudejans et al., 2002), and thus, of an optimal coupling between perception and action.

Klostermann et al. (2017) found a significant difference (i.e., increase) in relative quiet eye duration but not in absolute duration in the contested compared to the uncontested basketball shooting condition. As suggested in section “Introduction,” the change in relative quiet eye duration probably merely reflected a change in movement execution time (the jump phase was more than halved from around 1000 to around 400 ms) rather than in absolute quiet eye duration making it hard to draw conclusions about the effect of a defender on the visual control of the basketball jump shot. The current study is the first study showing that the proximity of a defender can reduce the absolute duration and worsen the timing of the final fixation on the rim.

In general, we cannot conclude that the proximity of a defender acts as a direct visual distraction for shooters that causes performance decrements. Although we found that both players with worse and with better performance fixated longer on the defender in the contested than in the uncontested condition, there was no significant difference between these groups. Furthermore, not all participants fixated on the defender in the contested condition, only some of them did. These fixations were of short duration and often occurred early in the progression from catch to ball release. Thus, if any, it seems that the proximity of a defender resulted in an indirect distraction for some of the players: their critical fixation on the rim became shorter and this was accompanied by reduced shooting accuracy.

Note that we did not analyze the final fixation on the rim relative to biomechanics of the shooting action (e.g., arm flexion time, ready position time, and arm extension time) other than ball release [i.e., the moment at which (visual) control of the ball ends]. It is therefore not possible to determine when during the arm movements of the shooting action the final fixation on the rim occurred. Speculating from the results of Oudejans and Coolen (2003) who reported a duration of the final extension movement of about 200 ms (for male shooters), it seems that the final fixation started prior to this movement partially overlapping it. Future research is needed to investigate this coupling in more detail. It is also important to mention that not finding an overall negative effect on shooting accuracy (as was found in earlier research and in the NBA, see section “Introduction”) may be related to the young age of the participants investigated. Although these players do belong to the talents of their age group, it is clear that they are still developing their skills. More research into the effects of a defender on shooting during different phases of development is needed as well as on the effects of the distance of the defender to the shooter as we now only investigated the two extremes of uncontested and contested shots.

Overall, the results of this study do indicate that our participants adapted their shooting movements to the proximity of a defender. This conclusion is consistent with those reported earlier in basketball (Rojas et al., 2000; Gorman and Maloney, 2016) and other sports (e.g., Rivilla-Garcia et al., 2011; Orth et al., 2014). For example, Rivilla-Garcia et al. (2011) showed that handball players adapted the ball velocity of the jump throw to the degree of opposition. However, we extended the existing literature by showing that some players were successful in these adaptations while others were not, and that this seemed to be related to their visual behavior. Players whose final fixations on the basket were affected in duration and timing showed a decrease in shooting accuracy, while players whose final fixations were unaffected did not show a decrease performance. Most important, the current study confirms that a direct opponent can change motor and gaze behavior of players in sport settings implying that it is essential to take this important constraint into account when creating representative tasks both for research and practice (see also Pinder et al., 2011, 2015). This is in line with the constraint-led approach advocated by Davids et al. (2008; Renshaw et al., 2010; Pinder et al., 2011), which takes its starting point in ecological psychology and the dynamical systems approach and the mutual relationship between performer and environment and the intricate coupling between perceiving and moving (Davids et al., 2008). In general, this implies that in investigating as well as training human movement, both the performer and the task should be embedded within the relevant constraints of the performance environment in order to obtain meaningful results.

### Practical Implications

Athletes have to invest many hours of practice to perform at a high level, also to accurately and consistently perform specific sport skills like the basketball jump shot. However, not only the quantity of practice but also the quality of practice is important. For many athletes and coaches, an important question is: how to design these training sessions? The results of the present study and of previous studies suggest that creating representative tasks is an important consideration. Small changes such as the proximity of a defender result in differences in movement execution and for some players in differences in gaze behavior. Therefore, it is essential to also train the basketball shot with a defender applying more or less defensive pressure as that may simulate the circumstances under which players shoot in games. Of course, the presence of defensive pressure is only one of the (many) relevant constraints that need to be taken into account into representative training designs. Some other constraints to consider are the action prior to the shot (Oudejans et al., 2012a), time constraints (Belling et al., 2015), the timing and duration of vision on the rim (Oudejans, 2012; Oudejans et al., 2012b), and distance to the rim (Elliott and White, 1989; Elliott, 1992). Thus, in sports practice, it is important to design tasks with constraints that are representative of the actual performance setting, and this can include the proximity of an opponent but also other factors like time or mental pressure.

## Ethics Statement

All subjects gave written informed consent in accordance with the Declaration of Helsinki. The protocol was approved by the Scientific and Ethical Review Committee of the Faculty of Behavioural and Movement Sciences of the Vrije Universiteit in Amsterdam.

## Author Contributions

MvM and RO contributed to the design of the study, data collection, and manuscript revision. MvM performed data analysis and wrote the first draft of the manuscript.

## Conflict of Interest Statement

The authors declare that the research was conducted in the absence of any commercial or financial relationships that could be construed as a potential conflict of interest.

## Footnotes

1 stats.nba.com
